# Supplementary material for: Active sites of copper-complex catalytic materials for electrochemical carbon dioxide reduction
Source: Nat Commun. 2018 Jan 29;9:415. doi: 10.1038/s41467-018-02819-7 (PMC5788987; doi:10.1038/s41467-018-02819-7)
Supplement: Supplementary file 1 — Supplementary Information [file 41467_2018_2819_MOESM1_ESM.pdf]

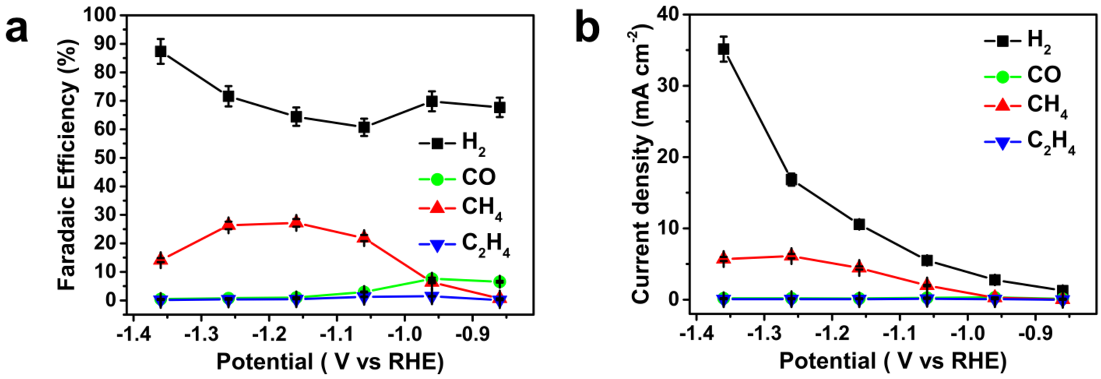

Supplementary Figure 1 | Electrocatalytic performance of HKUST-1 for CO_2 reduction. Potential-dependent **a** Faradaic efficiencies and **b** partial current densities of gas products.

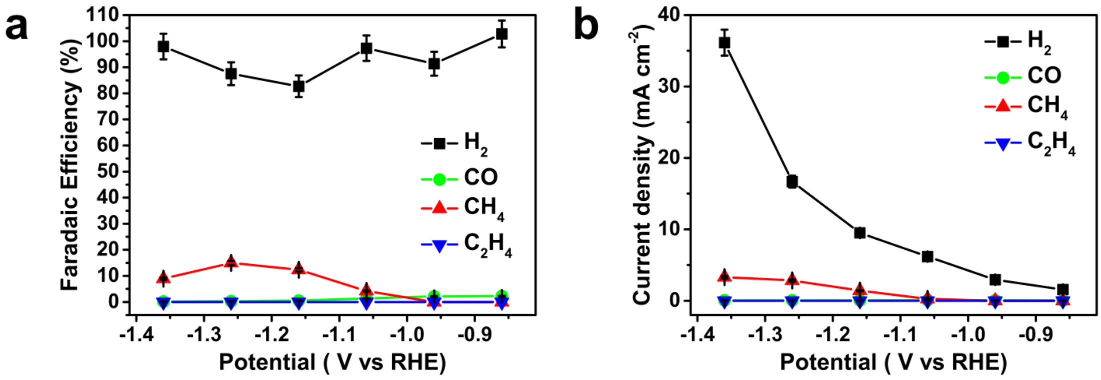

Supplementary Figure 2 | Electrocatalytic performance of [Cu(cyclam)]Cl_2 for CO_2 reduction. Potential-dependent **a** Faradaic efficiencies and **b** partial current densities of gas products.

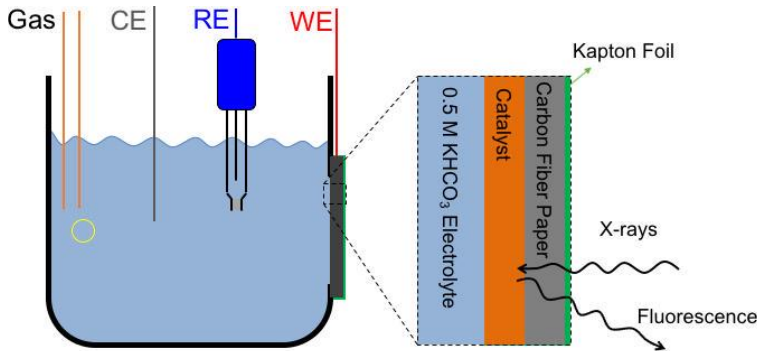

Supplementary Figure 3 | Schematic structure of the electrochemical cell used for *in-situ* XAS setup experiments. The catalyst material was deposited on the front side of the carbon fiber paper in contact with the CO_2-saturated 0.5 M $KHCO_3$ aqueous electrolyte. Kapton foil was applied on the back side of the carbon fiber paper facing the incident X-ray to avoid air entering the cell. CE, RE, and WE stand for counter, reference and working electrodes, respectively.

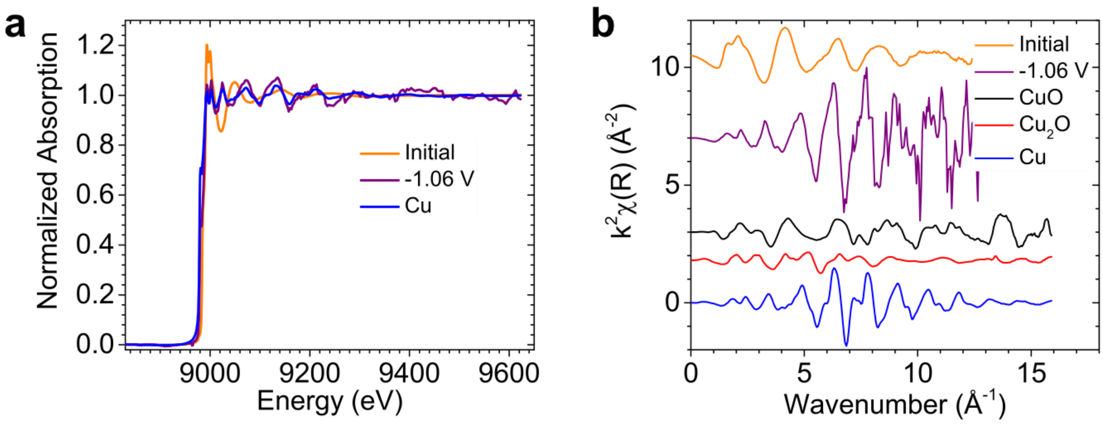

Supplementary Figure 4 | *In-situ* XAS measurements of [Cu(cyclam)]Cl_2 under electrochemical CO_2 reduction conditions. **a** XAS and **b** k-space EXAFS spectra. Cu metal formed at –1.06 V vs RHE detached from the electrode, causing much noise in the XAS spectrum and the EXAFS plot.

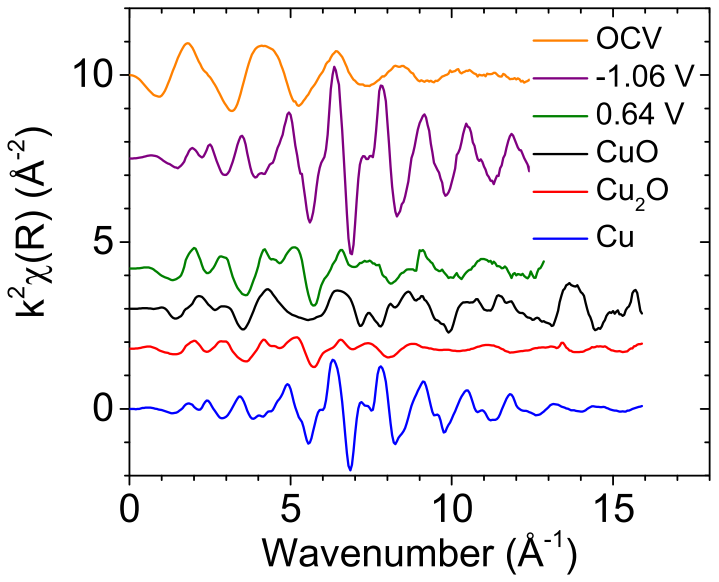

Supplementary Figure 5 | *In-situ* k-space EXAFS spectra of HKUST-1 under electrochemical CO_2 reduction conditions.

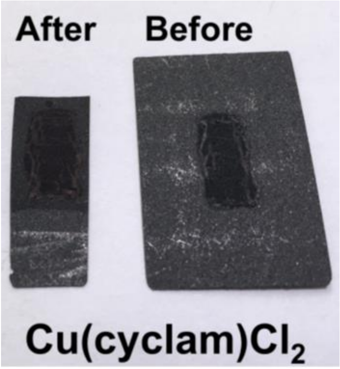

Supplementary Figure 6 | Photos of [Cu(cyclam)]Cl_2 catalyst electrodes before and after electrocatalysis.

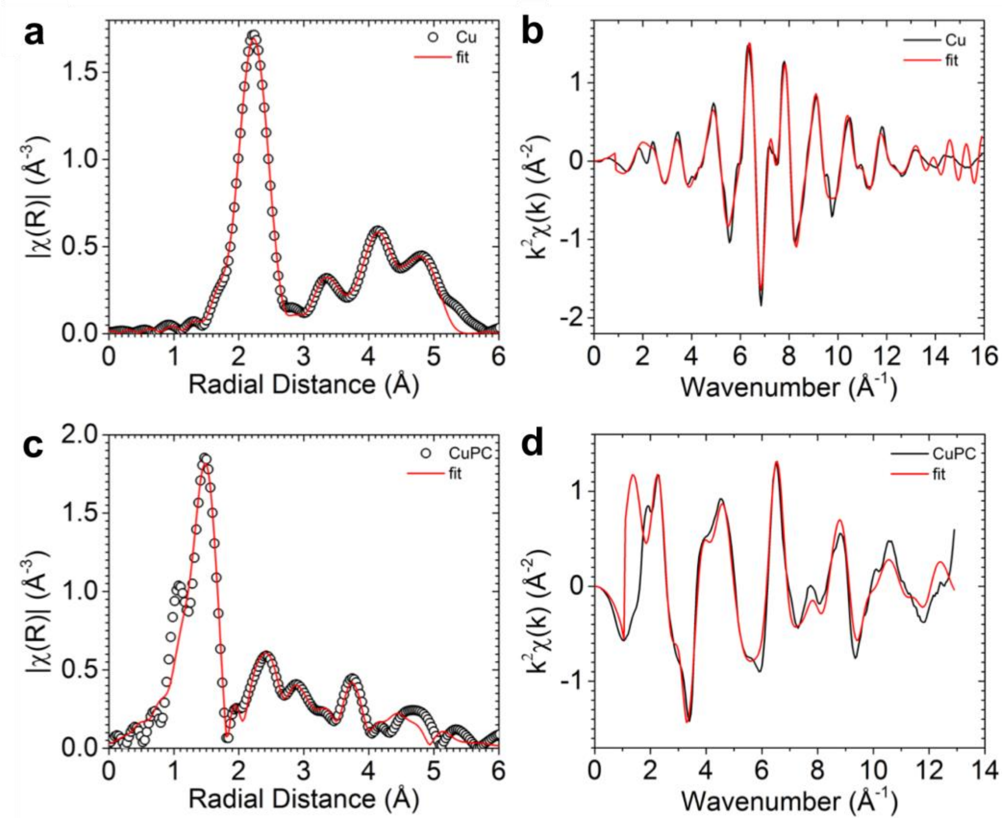

Supplementary Figure 7 | EXAFS fits for standards. a Cu metal R-space, **b** Cu metal k-space, **c** CuPc R-space, and **d** CuPc k-space.

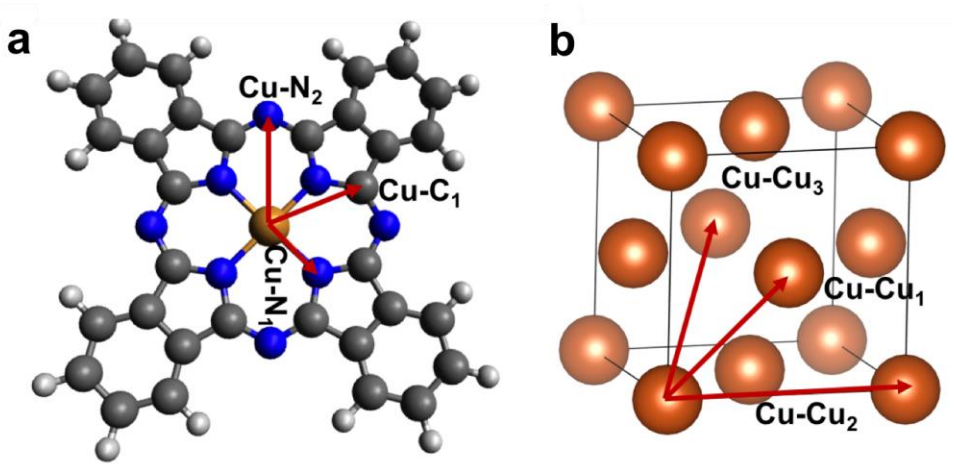

Supplementary Figure 8 | Atomic structures used for EXAFS fitting. a CuPc and **b** metallic Cu. The major scattering paths listed in Supplementary Table 1 are denoted here. Other scattering paths (e.g., multiple-scattering and fourth shell paths) used in fitting are not shown here. Color key: light grey - H; dark grey - C; blue - N; orange - Cu.

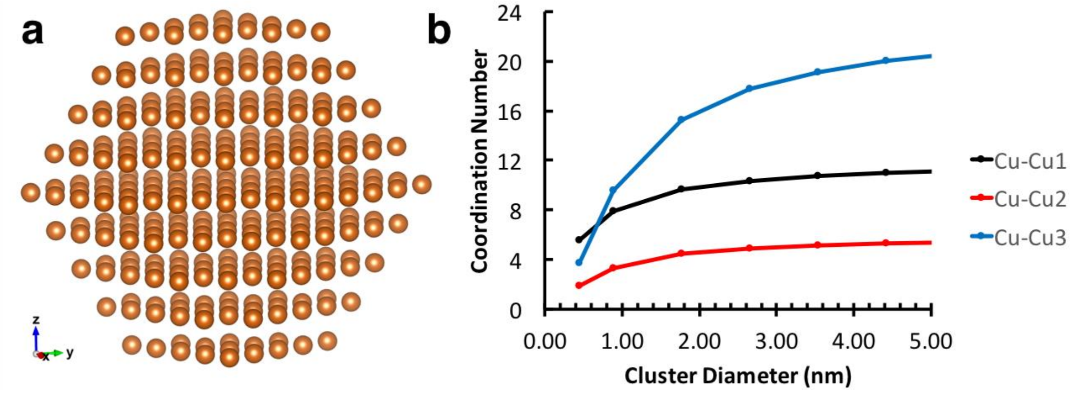

Supplementary Figure 9 | Cu nanocrystal model and its size-dependent Cu-Cu CNs for the first three shells of nearest neighbors. a A five-shell cuboctahedral Cu nanocrystal model. Areas shaded in blue and green represent (111)- and (100)-type planes, respectively. **b** Size-dependent Cu-Cu CNs for the first three shells of nearest neighbors for a cuboctahedral Cu nanocrystal.

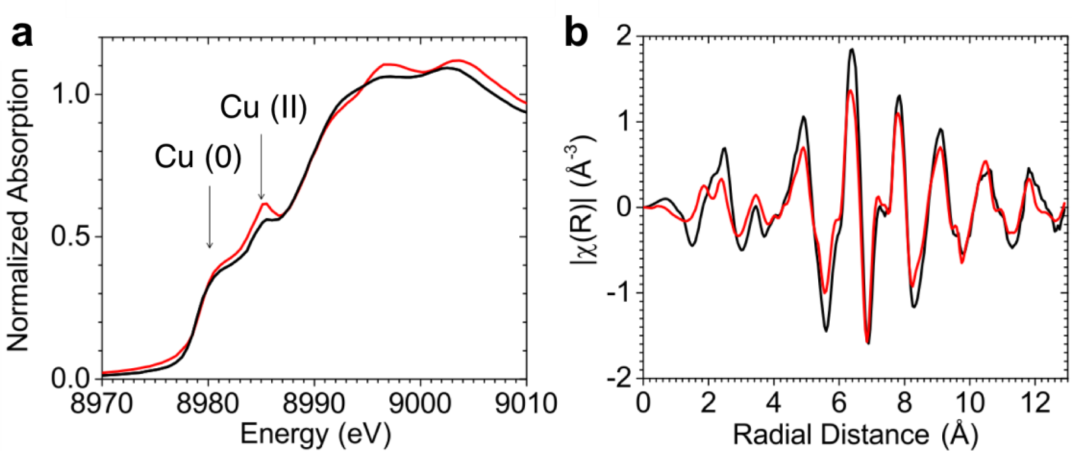

Supplementary Figure 10 | Linear combination fits of the XAS spectra of the CuPc catalyst at –1.06 V vs RHE using standard spectra of Cu foil and CuPc powder. **a** XANES and **b** EXAFS. The black lines are the measured data and the red lines are the fits.

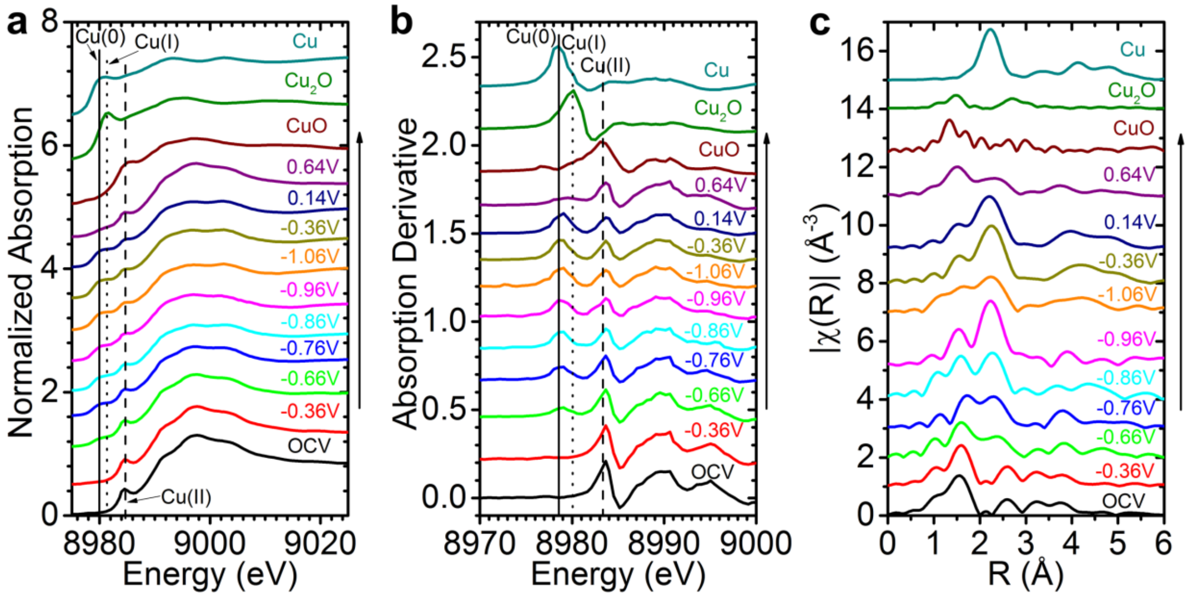

Supplementary Figure 11 | *In-situ* XAS measurements of copper(II)-5,10,15,20-tetrakis(2,6-dihydroxyphenyl)-porphyrin under electrocatalytic reaction conditions. a Cu K-edge XANES spectra, **b** first-order derivatives of the XANES spectra, and **c** Fourier-transformed Cu K-edge EXAFS spectra.

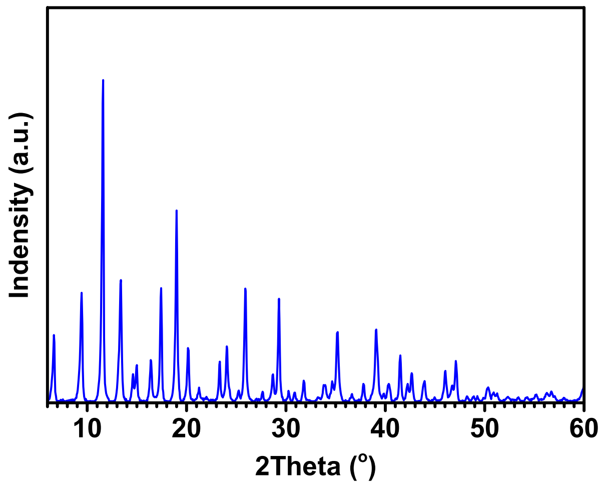

Supplementary Figure 12 | XRD pattern of as-synthesized HKUST-1 powder.

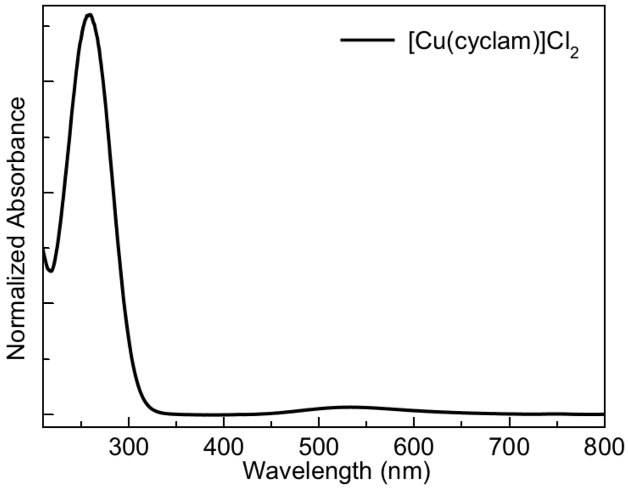

Supplementary Figure 13 | UV-Vis absorption spectrum of $[Cu(cyclam)]Cl_2$ in CH_3OH.

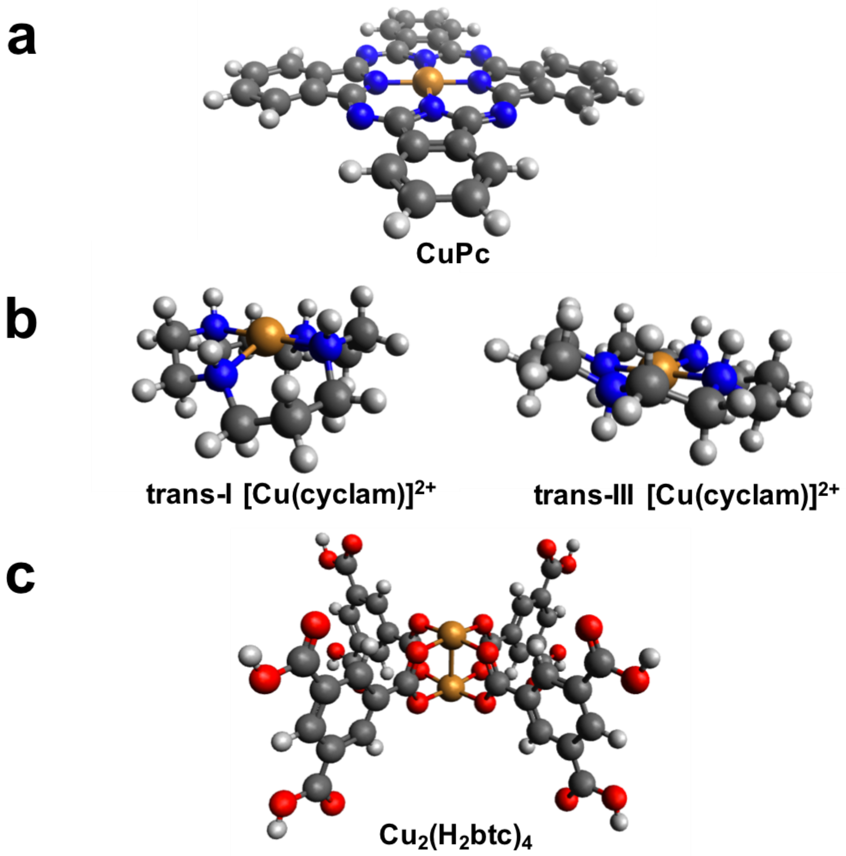

Supplementary Figure 14 | DFT-optimized structures. a CuPc, **b** $[Cu(cyclam)]^{2+}$ (trans-III and trans-I are two known isomers of metal cyclam complexes) and **c** $Cu_2(H_2btc)_4$ (a dimer unit of HKUST-1). Color key: light grey - H; dark grey - C; blue - N; red - O; orange - Cu.

Supplementary Table 1 | Fitting parameters of the co-refined EXAFS spectra of CuPc at different potentials (R: distance; σ^2: mean-square disorder; E_0: energy shift). The single digit numbers in parentheses for R are the last digit errors. The numbers in parentheses for CN are the full errors. The scattering paths (e.g., Cu-N_1 and Cu-Cu_1) are labeled in Supplementary Figure 8.

**Molecular CuPc**										
	Cu-N_1			Cu-C_1			Cu-N_2			
	CN	R (Å)	σ^2 (Å^2)	CN	R (Å)	σ^2 (Å^2)	CN	R (Å)	σ^2 (Å^2)	E_0 (eV)
Bulk	4	1.952	-	8	2.995	-	4	3.404	-	2.9(8)
OCV	4.0(0.5)	1.92(1)	0.005	5.2(1.3)	2.95(1)	0.002	4.0(0.5)	3.35(1)	0.011	
-0.36	3.8(0.4)	1.92(1)	0.005	5.1(0.8)	2.95(1)	0.002	3.8(0.4)	3.35(1)	0.011	
-0.66	2.1(0.3)	1.92(1)	0.005	3.5(0.8)	2.95(1)	0.002	2.1(0.3)	3.35(1)	0.011	
-0.76	1.9(0.3)	1.92(1)	0.005	3.3(0.8)	2.95(1)	0.002	1.9(0.3)	3.35(1)	0.011	
-0.86	1.6(0.3)	1.93(1)	0.005	1.8(0.9)	2.95(8)	0.002	-	-	-	
-0.96	1.3(0.4)	1.93(1)	0.005	2.4(1.1)	2.95(8)	0.002	-	-	-	
-1.06	1.2(0.5)	1.93(1)	0.005	2.6(1.5)	2.95(8)	0.002	-	-	-	
-0.36	1.2(0.5)	1.93(1)	0.005	2.6(1.5)	2.95(8)	0.002	-	-	-	
0.64	3.8(0.9)	1.92(1)	0.005	4.9(1.2)	2.95(1)	0.002	3.8(0.9)	3.35(1)	0.011	
**Cu Clusters**										
	Cu-Cu_1			Cu-Cu_2			Cu-Cu_3			
	CN	R (Å)	σ^2 (Å^2)	CN	R (Å)	σ^2 (Å^2)	CN	R (Å)	σ^2 (Å^2)	E_0 (eV)
Bulk	12	2.556	-	6	3.615	-	24	4.427	-	3.5(9)
OCV	-	-	-	-	-	-	-	-	-	
-0.36	-	-	-	-	-	-	-	-	-	
-0.66	0.4(0.3)	2.53(1)	0.003	0.6(0.9)	3.60(3)	0.003	-	-	-	
-0.76	0.8(0.4)	2.53(1)	0.003	0.7(0.9)	3.60(3)	0.003	-	-	-	
-0.86	4.5(0.5)	2.53(1)	0.003	1.1(1.3)	3.60(3)	0.003	17.5(17.5)	4.44(1)	0.007	
-0.96	5.9(0.6)	2.53(1)	0.003	1.4(1.4)	3.60(3)	0.003	14.2(3.9)	4.44(1)	0.007	

-1.06	6.5(0.9)	2.53(1)	0.003	1.4(2.0)	3.60(3)	0.003	15.8(5.6)	4.44(1)	0.007	
-0.36	6.5(0.9)	2.53(1)	0.003	1.4(2.0)	3.60(3)	0.003	15.8(5.6)	4.44(1)	0.007	
0.64	-	-	-	-	-	-	-	-	-	

Supplementary Table 2 | Calculated size-dependent CNs of the first three nearest neighbor shells for cuboctahedral Cu nanocrystals. The CNs of Cu-Cu_1, Cu-Cu_2, and Cu-Cu_3 for bulk Cu metal are 12, 6, and 24, respectively.

**Approx. Diameter (nm)**	**CN of Cu-Cu_1**	**CN of Cu-Cu_2**	**CN of Cu-Cu_3**
0.44	5.54	1.85	3.69
0.88	7.85	3.27	9.60
1.77	9.63	4.43	15.22
2.65	10.35	4.90	17.73
3.53	10.73	5.16	19.13
4.42	10.97	5.32	20.03
5.30	11.14	5.43	20.64
6.18	11.26	5.50	21.09
7.07	11.35	5.56	21.44
7.95	11.42	5.61	21.71
8.39	11.47	5.65	21.93

Supplementary Table 3 | Calculated free energies and standard reduction potentials of demetallation for CuPc, $Cu_2(H_2btc)_4$ (a dimer unit in HKUST-1), and $[Cu(cyclam)]^{2+}$ (trans-III and trans-I are two known isomers of metal cyclam complexes).

Species	ΔG^0 (kcal/mol)	$E^0_{CuL/Cu}$ (V vs SHE)
CuPc	52.7	-0.80
HKUST-1	27.9	-0.27
trans-III $[Cu(cyclam)]^{2+}$	41.7	-0.57
trans-I $[Cu(cyclam)]^{2+}$	36.2	-0.45

Supplementary Note 1 | Constructed thermodynamic pathways for reductive demetallation of: (A) CuPc, (B) $[Cu(cyclam)]^{2+}$ and (C) $Cu_2(H_2btc)_4$ (a dimer unit in HKUST-1).

(A)

$$\mathbf{CuPc(aq) + 2H^+(aq) + 2e^- \rightarrow Cu(s,bulk) + Pc^{2-}(aq)}$$

$$CuPc(aq) + 6H_2O(aq) + 2H^+(aq) \rightarrow H_2Pc\,(aq) + [Cu(H_2O)_6]^{2+}\,(aq)$$
$$[Cu(H_2O)_6]^{2+}(aq) + 2e^- \rightarrow Cu(s,bulk) + 6H_2O(aq)$$

(B)

$$\mathbf{[Cu(cyclam)]^{2+}(aq) + 2H^+(aq) + 2e^- \rightarrow Cu(s,bulk) + H_2cyclam^{2+}(aq)}$$

$$[Cu(cyclam)]^{2+}(aq) + 6H_2O(aq) + 2H^+(aq) \rightarrow H_2cyclam^{2+}\,(aq) + [Cu(H_2O)_6]^{2+}\,(aq)$$
$$[Cu(H_2O)_6]^{2+}(aq) + 2e^- \rightarrow Cu(s,bulk) + 6H_2O(aq)$$

(C)

$$\mathbf{{}^1/{}_2Cu_2(H_2btc)_4(aq) + 2H^+(aq) + 2e^- \rightarrow Cu(s,bulk) + 2H_3btc(aq)}$$

$${}^1/{}_2Cu_2(H_2btc)_4(aq) + 6H_2O(aq) + 2H^+(aq) \rightarrow H_3btc\,(aq) + [Cu(H_2O)_6]^{2+}\,(aq)$$
$$[Cu(H_2O)_6]^{2+}(aq) + 2e^- \rightarrow Cu(s,bulk) + 6H_2O(aq)$$

Supplementary Note 2 | Constructed thermodynamic pathway for re-metallation of H_2Pc.

$$\mathbf{H_2Pc(aq) + Cu(s,NP) \rightarrow H_2(g) + CuPc(aq)}$$

$$H_2Pc(aq) + Cu(g) \rightarrow H_2(g) + CuPc(aq)$$
$$Cu(s,NP) \rightarrow Cu(g)$$

Supplementary Note 3 | Synthetic route for $[Cu(cyclam)]Cl_2$.

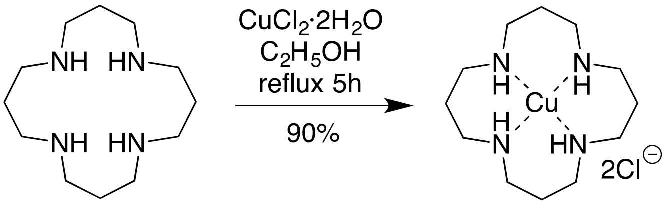

cyclam   [Cu(cyclam)]Cl2
